# Supplementary material for: A multi-step analysis and co-produced principles to support equitable partnership with Liverpool School of Tropical Medicine, 125 years on
Source: PLOS Glob Public Health. 2024 May 31;4(5):e0002091. doi: 10.1371/journal.pgph.0002091 (PMC11142479; doi:10.1371/journal.pgph.0002091)
Supplement: S1 Text — (DOCX) [file pgph.0002091.s002.docx]

**Supplementary File 1: LSTM Partnership Survey – 2021**

**Equitable partnerships: Amplifying perspectives from the countries where LSTM works**

**Introduction to the equitable partnership survey**

You have been invited to complete this survey because LSTM wants to understand how to better support equitable partnerships in the countries where it works. If you choose to participate, your views will be instrumental in shaping and informing LSTM’s practice and strategic vision in this area to ensure all our partnerships are of mutual benefit.

We have identified a number of topics for investigation (these are are adapted from the Research Fairness initiative domains of: fairness of opportunity; fair process; fair sharing of benefits, costs and outcomes) but please add any other issues you would like to tell us about, using the free-text boxes. You also have an opportunity to include your contact details at the end if you wish to be contacted for a follow up interview – however, if you wish to remain anonymous in the survey, but have a particular interest in being involved in an individual interview please email [rosie.steege@lstmed.ac.uk](mailto:rosie.steege@lstmed.ac.uk) or [robinson.karuga@lvcthealth.org](https://www.devex.com/news/global-fund-falls-short-of-18b-target-as-uk-italy-delay-pledges-104046) .

All data will be kept securely and limited members from [LSTM](https://www.elsevier.com/connect/open-access-creating-a-level-playing-field-for-the-global-south) & [LVCT Health](https://lvcthealth.org/) will have direct access to this. All your views expressed in this survey will be anonymous: we will anonymise data during analysis. We may analyse data by characteristics (e.g. gender, location of institution, age or type of activity of participant) but will ensure that in reporting no information that would reveal the identity of a respondent is used. By taking part you consent to us analysing this data.

We will report the findings of the survey to all LSTM partners through a report and also discuss the findings and potential ways to improve our partnership to be mutually beneficial. The survey should take no more than 30 minutes to complete.

**Section 1: Characteristics**

In this section, we will ask you about your personal characteristics.  **All questions in this section are optional.**

1. What is your age?

<30

30-<40

40-<50

50 - <60

≥60

prefer not to say

1. How do you define your gender? *If you prefer not to say, then please leave this question blank.*
2. What is your nationality? *If you prefer not to say, then please leave this question blank.*
3. Where is your organisation located?

Country:

1. What Is the highest level of academic qualification you have obtained?

Certificate/ Diploma

Bachelors

Masters

Doctorate

Other

1. What is the nature of your partnership with LSTM? *(Please type here)*
2. How many years have you personally been involved with LSTM?

< 2 years

2-<5 years

5-<10 years

10 -<20 years

≥20 years

1. How many years has your organisation been involved in partnership(s) with LSTM?

< 2 years

2-<5 years

5-<10 years

10 -<20 years

≥20 years

Don’t know

**Introduction to Section 2**

In the remainder of the survey, you will be asked your thoughts on your partnership with LSTM. The questions will focus on the following three domains:

- Fairness of opportunity
- Fair process
- Fair sharing of benefits, costs and outcomes

**Domain 1: Fairness of opportunity**

Increasing fairness of the opportunity that stakeholders have to influence research and education programmes at the stage or stages where it most impacts on their own ability to learn, contribute or participate, provides a sound foundation for respect in the current and future research partnerships. Fairness of opportunity sets the scene for the fair and efficient conduct of research / education and the fair and efficient sharing of costs and benefits later on. Partnerships with increasing respect for the interests and limitations of other partners last longer, work more efficiently, and create more resilience to overcome inevitable partnership stress productively.

1. **Who is the ‘main’/lead partner in your partnership?**
2. The following statements focus on **early engagement and research priorities.** Please select your response to each statement.

|  | Strongly disagree | Disagree | Neither agree nor disagree | Agree | Strongly agree |
| --- | --- | --- | --- | --- | --- |
| The research / education we are involved in with LSTM aligns to priorities or strategy of your organization |  |  |  |  |  |
| We were involved early in the partnership(s) and able to input into the aims, objectives, methods etc. for the partnership proposal(s) |  |  |  |  |  |
| We had equal opportunity to input into decision making at the early stages of the partnership development |  |  |  |  |  |
| Time was given to identify each partner’s strengths and weaknesses in expertise to deliver the project effectively |  |  |  |  |  |
| The roles within our partnership are clearly defined |  |  |  |  |  |
| There are clearly established processes for dispute resolution |  |  |  |  |  |
| At the start of our partnership our research / project management capacity was assessed and appropriate support discussed and identified |  |  |  |  |  |
| At the start of our partnership our contracting negotiation management capacity was assessed and appropriate support discussed and identified. |  |  |  |  |  |

1. Do you have any additional comments **on early engagement & project priorities** with LSTM?
2. The following statements focus on **financing arrangements.** Please select your response to each statement

|  | Strongly disagree | Disagree | Neither agree nor disagree | Agree | Strongly agree |
| --- | --- | --- | --- | --- | --- |
| LSTM’s own sources of financial support are well understood |  |  |  |  |  |
| Determinants of LSTM’s capability (both limitations and capacity) to provide financial support are well understood |  |  |  |  |  |
| There is equity in financial allocation across the partnership |  |  |  |  |  |
| There is equity in non-financial contributions across the partnership |  |  |  |  |  |

1. Do you have any additional comments on **financing arrangements** with LSTM? *Please type your response in the box below*

**Domain 2: Fair process**

Domain 2 aims to improve fairness in how research / education is conducted and research /education partnerships and programmes are implemented. Expectations of different partners are usually different. By creating clarity in how organisations deal with these challenges in principle and in practice, research stakeholders can reduce misunderstandings and can increase the capacity of all partners to live up to the expectations that others may have of them.

1. The following statements focus on **hiring, capacity building and sourcing.** *Please select your response to each statement*

|  | Strongly disagree | Disagree | Neither agree nor disagree | Agree | Strongly agree |
| --- | --- | --- | --- | --- | --- |
| Hiring local staff is encouraged in the partnership(s) with LSTM |  |  |  |  |  |
| There are clear criteria for involving LSTM staff in the partnership you have with LSTM |  |  |  |  |  |
| The partnership with LSTM includes a commitment to capacity building within my organisation |  |  |  |  |  |
| The partnership with LSTM provides resources for capacity building of project staff |  |  |  |  |  |
| The partnership with LSTM provides resources for training and higher education of project management staff |  |  |  |  |  |
| The partnership with LSTM supports partners to become better able to access competitive grants |  |  |  |  |  |
| Partnership with LSTM is helpful in assisting me to build my organisation’s capacity to influence policy and practice **locally.** |  |  |  |  |  |
| Partnership with LSTM is helpful in assisting my organisation’s capacity to influence policy and practice **globally.** |  |  |  |  |  |

1. Do you have any additional comments **hiring, capacity building or sourcing** with LSTM? *Please add them here or explain any answers above*

**Ethical and Impactful Research**

1. The following statements focus on **ethical and impactful research / education.** *Please select your response to each statement.*

|  | Strongly disagree | Disagree | Neither agree nor disagree | Agree | Strongly agree |
| --- | --- | --- | --- | --- | --- |
| There is respect and understanding in discussion and decisions around ethical review in our partnership with LSTM |  |  |  |  |  |
| Our partnership with LSTM includes processes to support local research ethics review capacity if required (e.g. by sourcing independent expertise to conduct ethical review)? |  |  |  |  |  |

1. Do you have any additional comments about **ethical and impactful research / education**? If so, please add them here or explain any answers above

**Data sharing and Intellectual Property (IP)**

|  | Strongly disagree | Disagree | Neither agree nor disagree | Agree | Strongly agree |
| --- | --- | --- | --- | --- | --- |
| Data sharing agreements are in place |  |  |  |  |  |
| There was opportunity to input into these |  |  |  |  |  |
| There is an agreement on intellectual property rights use that I am comfortable with |  |  |  |  |  |
| The partnership with LSTM includes explicit pre- / post-research discussions and negotiations with partners concerning the sharing of IP |  |  |  |  |  |
| Any disagreements on IP were dealt with fairly |  |  |  |  |  |
| The partnership with LSTM includes support for IP contracting |  |  |  |  |  |
| The partnership with LSTM includes access to technology for my organisation |  |  |  |  |  |

1. Do you have any additional comments about **data sharing or IP**? If so, please add them here, or explain any answers above

**Budgeting and Financial Management**

|  | Strongly disagree | Disagree | Neither agree nor disagree | Agree | Strongly agree |
| --- | --- | --- | --- | --- | --- |
| The partnership with LSTM includes support with preparation and management of budgets if required |  |  |  |  |  |
| The partnership with LSTM included, at the start, assessment of the competency of my organisation with regards to financial management for research |  |  |  |  |  |

1. Do you have any additional comments about **budgeting and financial management** in your partnership with LSTM? If so, please add them here or please explain any answers above

**Domain three: Fair sharing of benefits, costs and outcomes**

Domain 3 deals with improving fairness in sharing the costs, benefits and outcomes of research / education. It focuses both on short-term costs, benefits and outcomes of individual studies / education programmes, but also on the medium- and long-term impact that partnership can have on the ability of partners to grow their own capacity, increase their ability to compete in attracting funding, on social impact, and on future economic benefits of research in terms of economic activity, technology sector growth, and both technical and social innovations benefits accruing to all in the partnership.

1. The following statements focus on **innovation.** Please select your response to each statement.

|  | Strongly disagree | Disagree | Neither agree nor disagree | Agree | Strongly agree |
| --- | --- | --- | --- | --- | --- |
| The partnership with LSTM gives scope for us to be innovative from the inception of projects |  |  |  |  |  |
| The partnership with LSTM includes, in the research contract negotiations and in research partnership agreements, clear statements on how future spin-off economic activities resulting from the research will be shared |  |  |  |  |  |
| The partnership with LSTM facilitates institutional or national discussions on this matter – supporting partners to make sure that research does not end with publications |  |  |  |  |  |

1. Do you have any additional comments about **innovation** in your partnership with LSTM? If so, please add them here or please explain any answers above
2. The following statements focus on **culture and inclusivity.** Please select your response to each statement.

|  | Strongly disagree | Disagree | Neither agree nor disagree | Agree | Strongly agree |
| --- | --- | --- | --- | --- | --- |
| The partnership with LSTM encourages diversity of participation in research and in the partnership by gender, age, ethnicity and socio-economic status? |  |  |  |  |  |
| The partnership with LSTM works to assess, report and minimise environmental impact of research partnerships |  |  |  |  |  |
| The partnership with LSTM encourages/supports my organisation to create a culture of fairness in its research partnerships |  |  |  |  |  |
| The partnership with LSTM attempts to ensure that research funder or sponsor demands do not create unfairness in partnerships |  |  |  |  |  |
| The relationships between people in our partnership are respectful |  |  |  |  |  |
| The decisions made in our partnership are equitable |  |  |  |  |  |
| I plan to collaborate with LSTM in the future |  |  |  |  |  |

1. Do you have any additional comments about **culture and inclusivity** in your partnership with LSTM? If so, please add them here or please explain any answers above

**Research uptake**

1. The following statements focus on **research uptake**, meaning the use of research by others including researchers and policy makers. Please select your response to each statement.

|  | Strongly disagree | Disagree | Neither agree nor disagree | Agree | Strongly agree |
| --- | --- | --- | --- | --- | --- |
| The Partnership with LSTM is helpful in enabling my organisation to have more interactions with policy makers and other stakeholders **locally** |  |  |  |  |  |
| The partnership with LSTM is helpful in enabling my organisation to have more interactions with policy makers and other stakeholders **globally** |  |  |  |  |  |
| Publishing authorship is usually agreed collaboratively and in an equitable way in the partnership with LSTM |  |  |  |  |  |

1. Please explain your answers above

**Responsiveness during the COVID-19 pandemic**

1. We would also like your thoughts on how the LSTM has responded to the COVID-19 pandemic. Please select your response to this statement.

|  | Strongly disagree | Disagree | Neither agree nor disagree | Agree | Strongly agree |
| --- | --- | --- | --- | --- | --- |
| The partnership with LSTM has provided support during the COVID-19 pandemic |  |  |  |  |  |

1. Please explain your answer above
2. Do you have any additional comments about the **responsiveness of the partnership with LSTM to COVID-19**? If so, please add them here.

**Final thoughts**

1. Do you have any suggestions for improving partnership with LSTM? If so, please write them here.
2. Is there anything you think partnership with LSTM does well and you would like to see continue?
3. Finally, is there anything you would like to add that hasn't been covered in the survey?

**Many thanks for taking the time to complete this survey.**

**Would you like to include your email for contact?**

Appendix 2: Abridged anonymised survey responses (does not include free text responses)


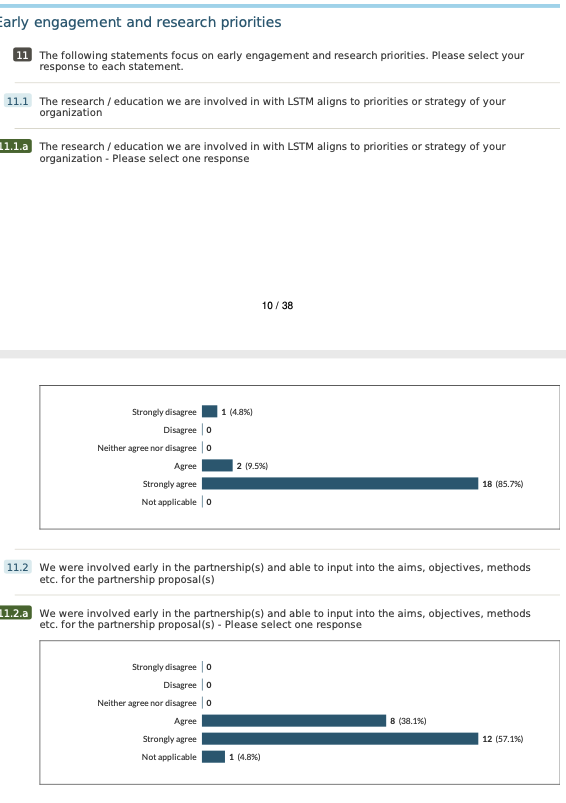


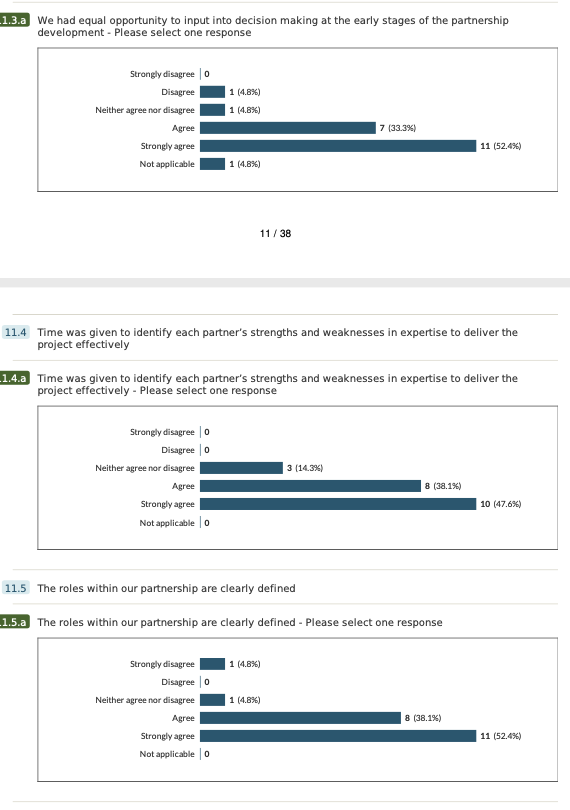


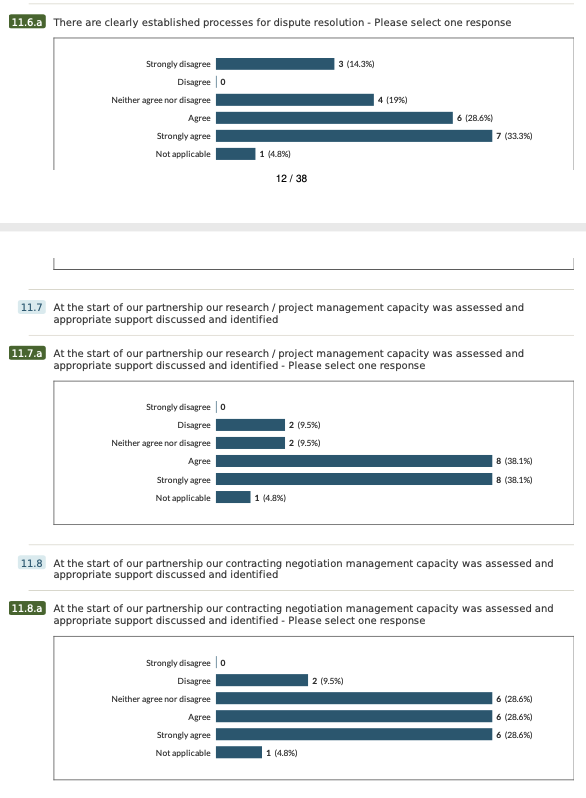


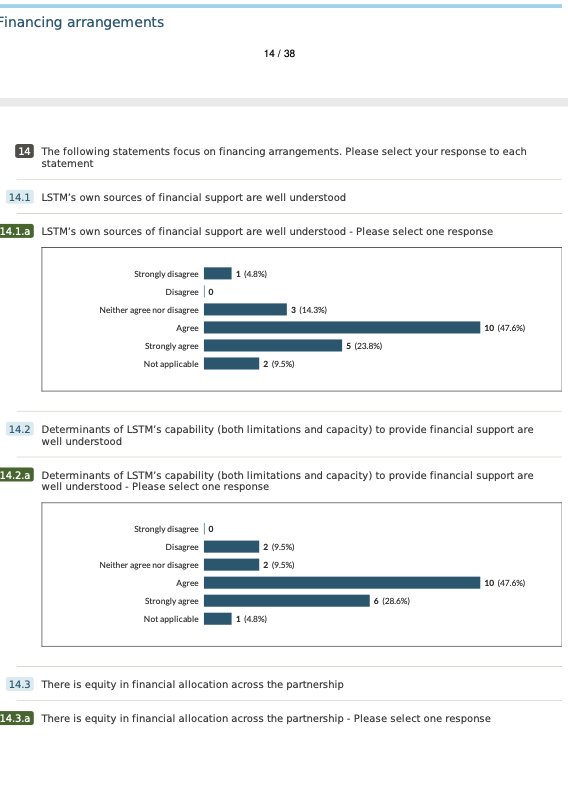


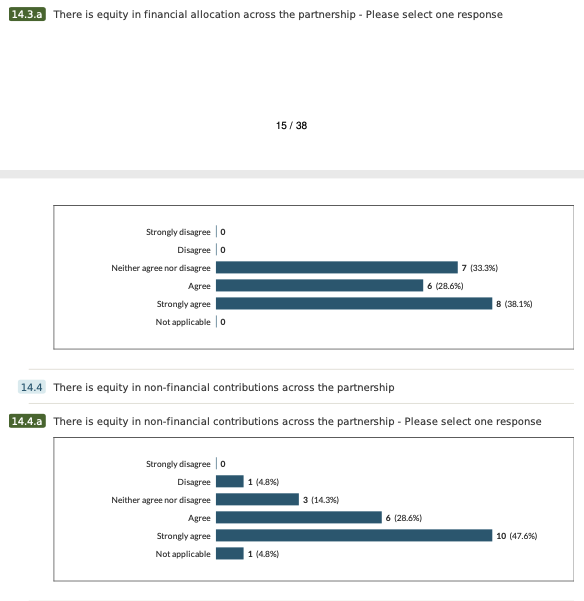


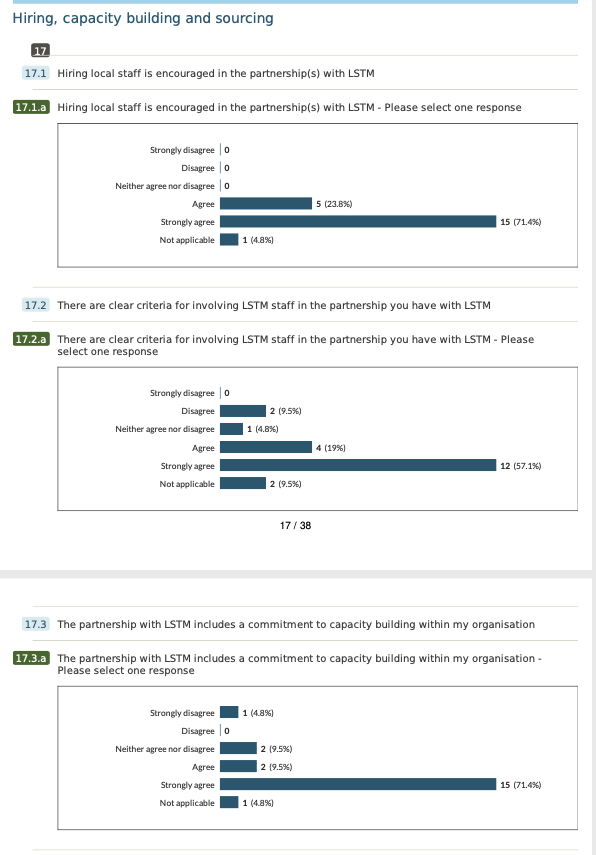


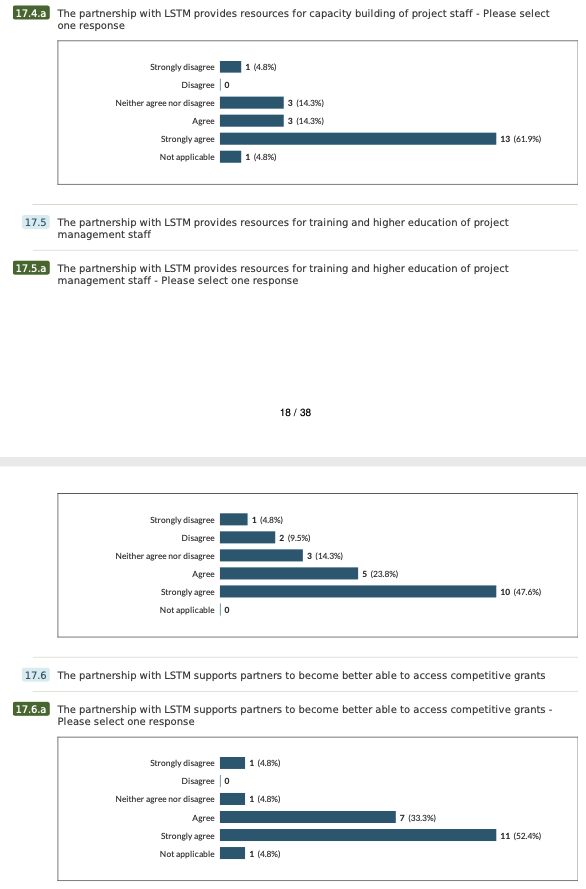


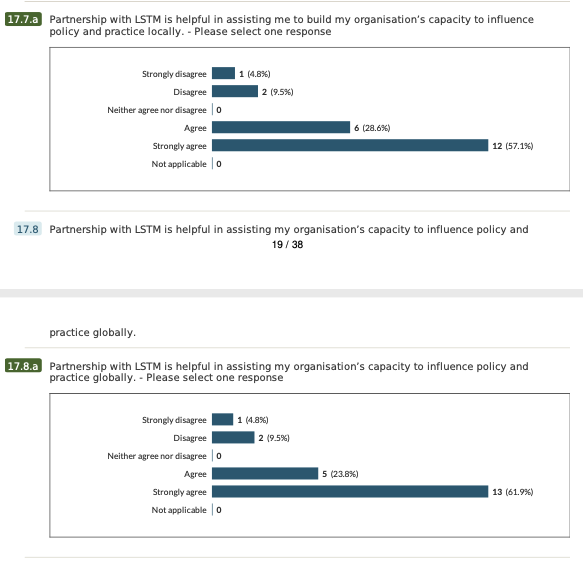


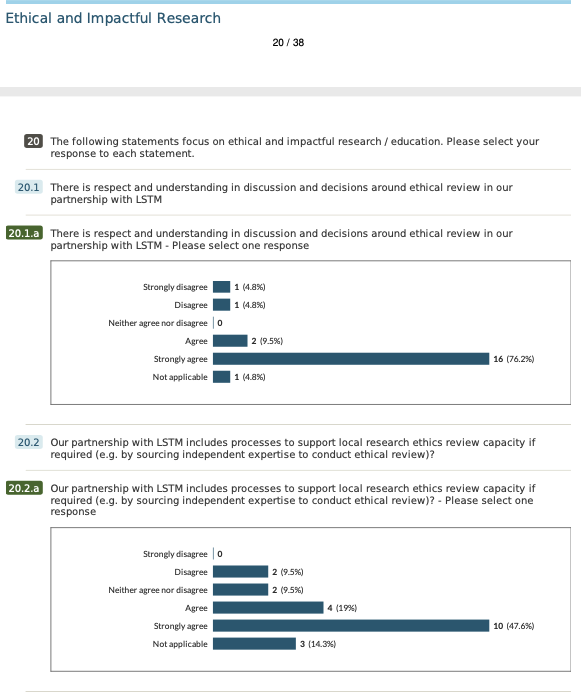


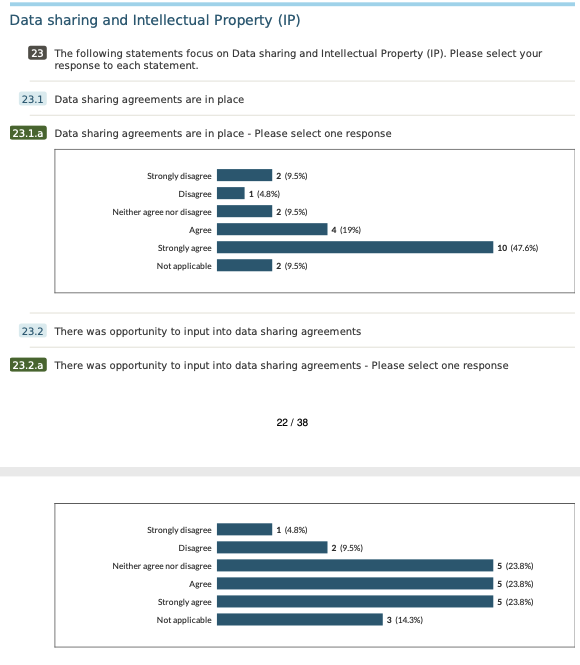


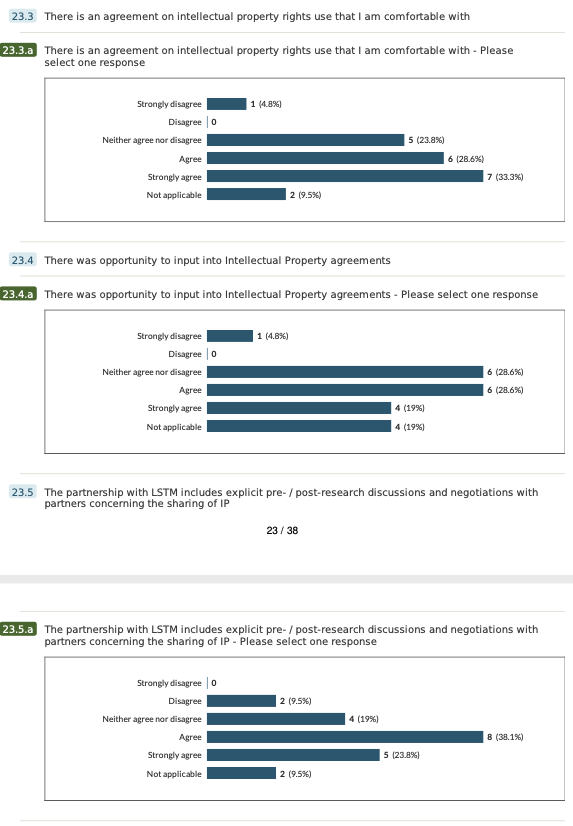


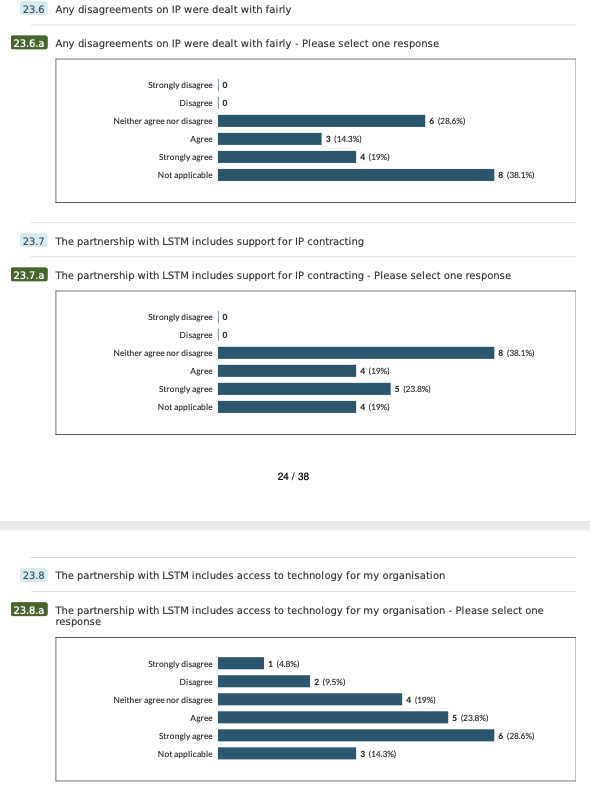


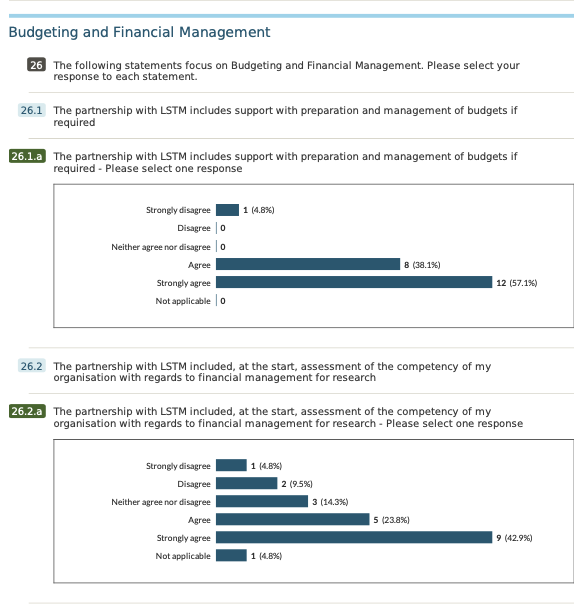


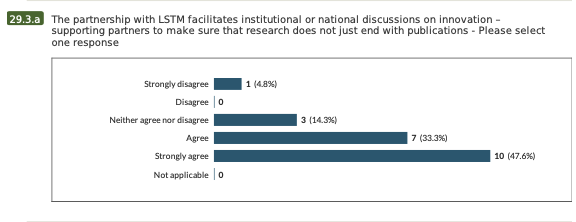


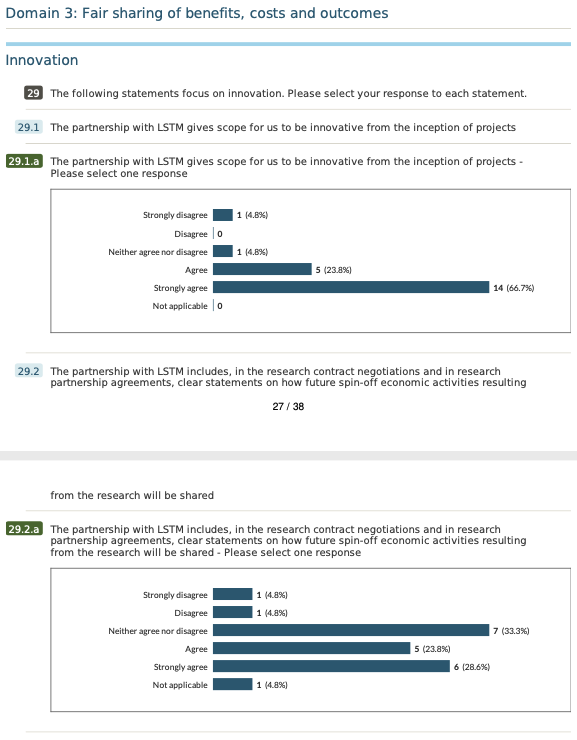


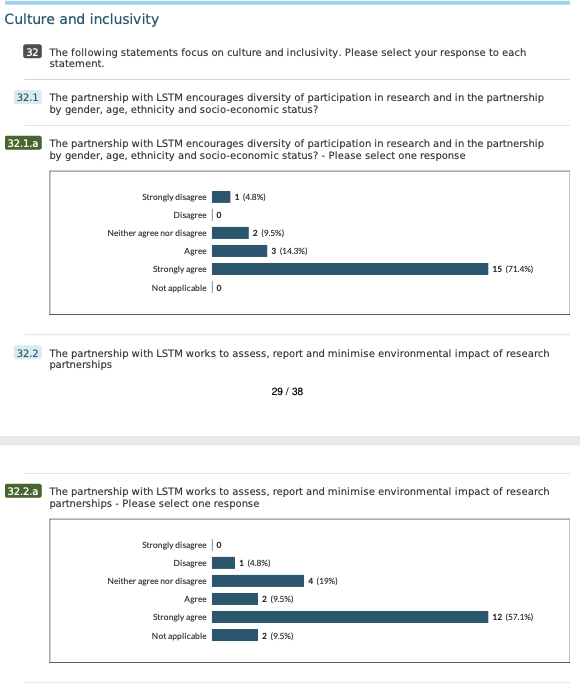


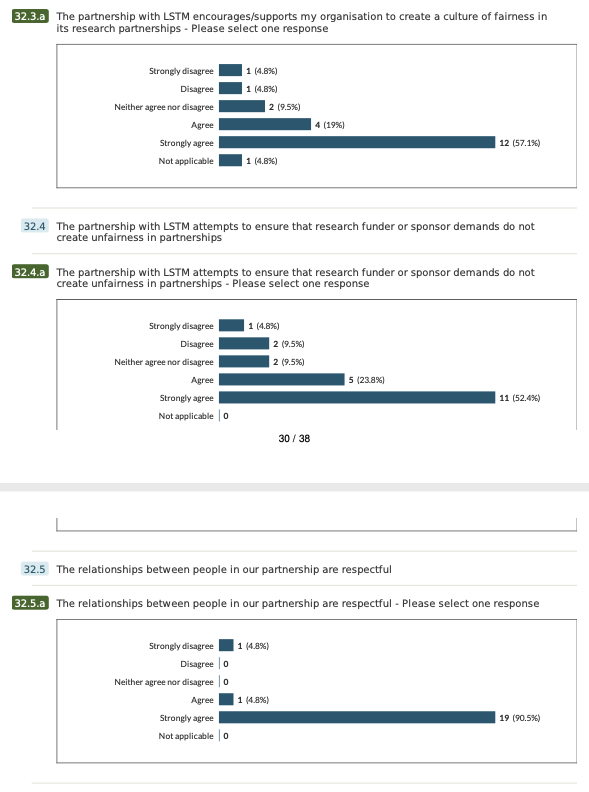


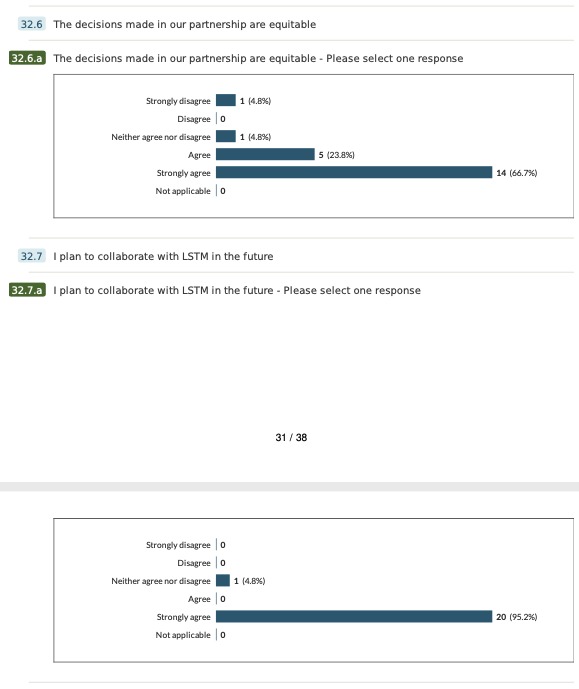


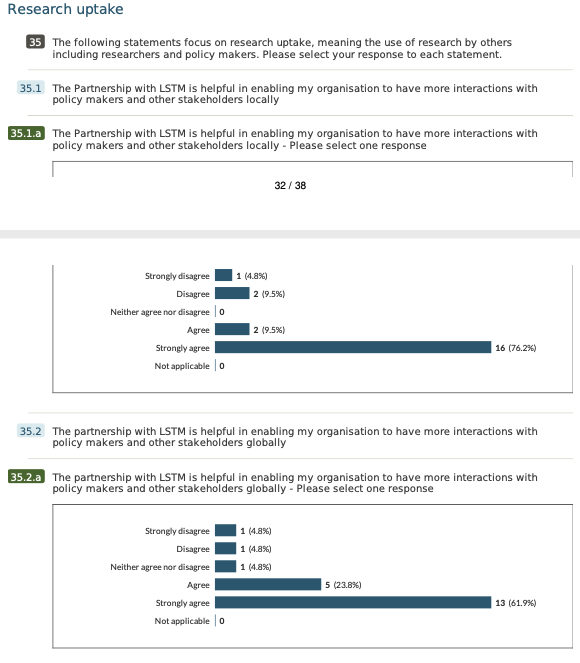


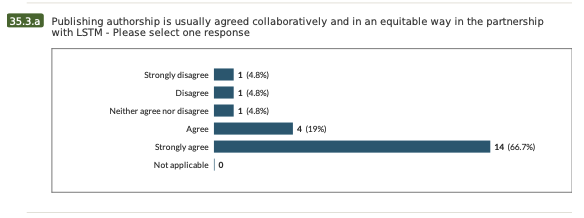


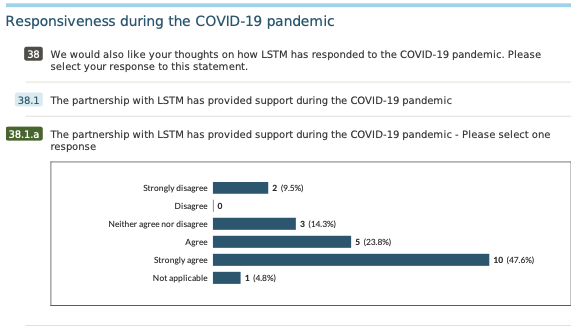


Appendix 3: **Topic Guide for Key Informant Interviews**

**Equitable partnerships: Amplifying perspectives from partner organisations in countries where LSTM works**

**Introductory questions**

1. Please tell me more about yourself and your organisation
2. How have you and LSTM collaborated?
   1. *Probe: How long for each partnership*
   2. *Probe: What is the nature of the collaboration – research, education, other?*
   3. *Probe has it changed through time?*
3. What words would you use to describe the partnership?
4. When referring to your country context, what language are you most comfortable with? E.g. would you have any preference between the use of the phrases “Global South” and LMIC settings? Why?

**Agenda setting**

1. Please describe to me how the agenda/research questions / education priorities for your partnership was/ were developed
   1. *What was the process?*
   2. *Probe the process of developing the agenda / research questions / education priorities for each partnership (if multiple)*
   3. *What was your role in proposal development?*
      1. *Probe - How were your views incorporated?*
      2. *Probe - How did that influence the partnership?*
      3. *Probe - space for innovation from both LSTM and partners based in L/MICs/global south?*
      4. *Probe - Engagement of local knowledge systems?*
2. How did these agenda / research questions / education priorities respond to needs in your context?
   1. *In what ways can partnership with LSTM support locally- driven priorities?*
3. Do you feel that the partnership plays to your strengths as an organization - How?
   1. *Probe: Are the teams skillset recognized and valued?*
   2. *Dynamics of HIC vs LMIC expertise*
4. How are power differentials in the partnership discussed / made explicit / negotiated*?*

**Impacts and outputs**

1. What are the key outputs that were developed as a result of your partnership with LSTM?
   1. *Probe for each partnership, if multiple*
2. What are your views about the distribution of authorship between LSTM and your organization in the different outputs from your partnership with LSTM
   1. *Probe for views about each partnership*
3. What is the approach to disseminating lessons learnt / best practices developed in your partnership with LSTM?
   1. *Probe for local and global dissemination – explicit role of partners based in middle and low income countries*
   2. *Who takes lead role?*
4. What would you say have been the impact of the collaboration between LSTM and your organization?
   1. Probe: On broader development in your context
   2. Probe: On policy changes in your context
   3. Probe: For communities
5. What are your views about capacity strengthening in the partnership with LSTM?
   1. *Probe for specific examples of capacity strengthening*
   2. *Institutional capacity strengthening*
   3. *Individual capacity strengthening*

**Future of partnership**

1. What are some things that you can recommend for LSTM to strengthen to make the current and future partnerships more mutually beneficial?
2. In your own words, how would you define equitable partnerships between Global Northern/HIC and Global Southern/LMIC institutions?
   1. How do you think this could be evaluated?
3. How does your description of equitable partnerships relate to your current partnership(s) with LSTM?
   1. Probe: diversity of participation in research by gender, age, ethnicity and socio-economic status?
4. From your experience partnering with LSTM, what would you say are the principles of equitable partnership?
   1. How would you suggest holding LSTM accountable to equitable partnerships?
   2. How would you suggest holding your organisation accountable to equitable partnerships?
   3. If you had a concern about the partnership – how would you voice/ address that?

**Funding**

1. How would you say the funding structure influences your partnership?
   1. *What are the major sources of funding for projects in your partnership with LSTM*
   2. *Does funding for projects in your partnership always flow from North to South?*
   3. *If not, what are examples of some sources of funding in LMICs?*
   4. *Who decides on budget allocations?*
2. In the environment of funding cuts to the UK aid budget. Has this changed the dynamic of the partnership?
   1. *How do you think LSTM and the partnership has handled the situation?*
   2. *What could LSTM and the partnership do to best support you/ be an effective ally in this moment?*

Closing -

1. Is there anything you want to flag that LSTM does particularly well/ not so well at in fostering mutually beneficial partnerships?

Thank you for your time. Do you have anything else you would like to share?

Appendix 4:

**Consensus statement** **as per Morton et al. guidance on equitable authorship:**

1. **How does the study address local research and policy priorities?**

Our study was specifically designed to support equity within transboundary partnerships. Supporting equitable partnerships is a key component of decolonising global health which is an issue of major priority in global health partnerships.

1. **How were local researchers involved in study design?**

This was a global study designed by researchers in LSTM Liverpool (BS, ST, RS, SC) and LVCT health Kenya (RK and LO). LVCT Health and LSTM are longstanding partners and both are aligned in their goals to support equity in partnership. The study was designed to engage the perspectives of transboundary partners to co-develop principles for equitable partnerships in this area.

1. **How has funding been used to support the local research team?**

The funding was used for pay for staff time in both LVCT Health and LSTM.

1. **How are research staff who conducted data collection acknowledged?**

RS and RK conducted the data collection for the survey and KIIs, SC was also involved in data collection for the workshop. All research staff involved are authors on this paper.

1. **Do all members of the research partnership have access to study data?**

All members of the partnership have access to data.

1. **How was data used to develop analytical skills within the partnership?**

Most of the members of the research team are mid-career or senior researchers. SC is a research assistant and doctoral candidate and played a key role in workshop facilitation and analysis. She was supported by RS and RK. SC also presented on behalf of the research team at the Health Systems Global Conference in Bogotá 2022, in a collaborative session on equitable partnerships.

1. **How have research partners collaborated in interpreting study data?**

LVCT health colleagues (RK, LO) played a key role in analysing and interpreting the study data. In addition:

- All participants were invited to a virtual workshop where the findings were presented for feedback followed by a process of co-creating principles. The principles were then also shared by email with all partners for any further additions or reflections.
- The study process, feedback and principles were also fed back to LSTM staff at a workshop and anonymous feedback was elicited through easy retro
- The study process, feedback and principles were also shared as part of a session on “Equitable Partnerships” held at the Health Systems Global conference in Bogota in November, 2022 and discussed in a world café format.
- Partners were also given opportunity to comment on the manuscript before submission

1. **How were research partners supported to develop writing skills?**

We supported each other in the development of the outputs – including ethical protocols, PowerPoints and papers.

1. **How will research products be shared to address local needs?**

We have opted for open access publication so that our research process and findings and principles are open for all to learn from and adapt as appropriate. These principles can be used by partners to hold LSTM to account in future collaborations.

1. **How is the leadership, contribution and ownership of this work by LMIC researchers recognised within the authorship?**

The contribution of LVCT in shaping and conducting this research and output has been recognised with joint first (RK) and last authorship (LO) positions.

1. **How have early career researchers across the partnership been included within the authorship team?**

We have included SC as a doctoral researcher within the authorship team, her critical role has been acknowledged as a corresponding author.

1. **How has gender balance been addressed within the authorship?**

Four authors are women (RS, SC, ST, LO) and two men (RK, BS). We have women as both first author (RS) and last (LO/ST) author positions.

1. **How has the project contributed to training of LMIC researchers?**

The LMIC researchers involved in the research are mid-senior in their career so this was not applicable, although the whole research team (global south and north) strengthened their capacity through the joint research process.

1. **How has the project contributed to improvements in local infrastructure?**

This project has not directly contributed to improvements in local infrastructure.

1. **What safeguarding procedures were used to protect local study participants and researchers?**

We used the [LSTM safeguarding policy](https://writingonthewall.org.uk/wp-content/uploads/2022/05/04_Stephen-Small_Liverpool-School-of-Tropical-Medicine_V2-1.pdf) to guide this study; no safeguarding issues emerged during the study.
