# Supplementary material for: A multi-step analysis and co-produced principles to support equitable partnership with Liverpool School of Tropical Medicine, 125 years on
Source: PLOS Glob Public Health. 2024 May 31;4(5):e0002091. doi: 10.1371/journal.pgph.0002091 (PMC11142479; doi:10.1371/journal.pgph.0002091)
Supplement: S2 Text — (DOCX) [file pgph.0002091.s003.docx]

**S3: Topic Guide for Key Informant Interviews**

**Equitable partnerships: Amplifying perspectives from partner organisations in countries where LSTM works**

**Introductory questions**

1. Please tell me more about yourself and your organisation
2. How have you and LSTM collaborated?
   1. *Probe: How long for each partnership*
   2. *Probe: What is the nature of the collaboration – research, education, other?*
   3. *Probe has it changed through time?*
3. What words would you use to describe the partnership?
4. When referring to your country context, what language are you most comfortable with? E.g. would you have any preference between the use of the phrases “Global South” and LMIC settings? Why?

**Agenda setting**

1. Please describe to me how the agenda/research questions / education priorities for your partnership was/ were developed
   1. *What was the process?*
   2. *Probe the process of developing the agenda / research questions / education priorities for each partnership (if multiple)*
   3. *What was your role in proposal development?*
      1. *Probe - How were your views incorporated?*
      2. *Probe - How did that influence the partnership?*
      3. *Probe - space for innovation from both LSTM and partners based in L/MICs/global south?*
      4. *Probe - Engagement of local knowledge systems?*
2. How did these agenda / research questions / education priorities respond to needs in your context?
   1. *In what ways can partnership with LSTM support locally- driven priorities?*
3. Do you feel that the partnership plays to your strengths as an organization - How?
   1. *Probe: Are the teams skillset recognized and valued?*
   2. *Dynamics of HIC vs LMIC expertise*
4. How are power differentials in the partnership discussed / made explicit / negotiated*?*

**Impacts and outputs**

1. What are the key outputs that were developed as a result of your partnership with LSTM?
   1. *Probe for each partnership, if multiple*
2. What are your views about the distribution of authorship between LSTM and your organization in the different outputs from your partnership with LSTM
   1. *Probe for views about each partnership*
3. What is the approach to disseminating lessons learnt / best practices developed in your partnership with LSTM?
   1. *Probe for local and global dissemination – explicit role of partners based in middle and low income countries*
   2. *Who takes lead role?*
4. What would you say have been the impact of the collaboration between LSTM and your organization?
   1. Probe: On broader development in your context
   2. Probe: On policy changes in your context
   3. Probe: For communities
5. What are your views about capacity strengthening in the partnership with LSTM?
   1. *Probe for specific examples of capacity strengthening*
   2. *Institutional capacity strengthening*
   3. *Individual capacity strengthening*

**Future of partnership**

1. What are some things that you can recommend for LSTM to strengthen to make the current and future partnerships more mutually beneficial?
2. In your own words, how would you define equitable partnerships between Global Northern/HIC and Global Southern/LMIC institutions?
   1. How do you think this could be evaluated?
3. How does your description of equitable partnerships relate to your current partnership(s) with LSTM?
   1. Probe: diversity of participation in research by gender, age, ethnicity and socio-economic status?
4. From your experience partnering with LSTM, what would you say are the principles of equitable partnership?
   1. How would you suggest holding LSTM accountable to equitable partnerships?
   2. How would you suggest holding your organisation accountable to equitable partnerships?
   3. If you had a concern about the partnership – how would you voice/ address that?

**Funding**

1. How would you say the funding structure influences your partnership?
   1. *What are the major sources of funding for projects in your partnership with LSTM*
   2. *Does funding for projects in your partnership always flow from North to South?*
   3. *If not, what are examples of some sources of funding in LMICs?*
   4. *Who decides on budget allocations?*
2. In the environment of funding cuts to the UK aid budget. Has this changed the dynamic of the partnership?
   1. *How do you think LSTM and the partnership has handled the situation?*
   2. *What could LSTM and the partnership do to best support you/ be an effective ally in this moment?*

Closing -

1. Is there anything you want to flag that LSTM does particularly well/ not so well at in fostering mutually beneficial partnerships?

Thank you for your time. Do you have anything else you would like to share?
