# Supplementary figures and images for: A multi-step analysis and co-produced principles to support equitable partnership with Liverpool School of Tropical Medicine, 125 years on
Source: PLOS Glob Public Health. 2024 May 31;4(5):e0002091. doi: 10.1371/journal.pgph.0002091 (PMC11142479; doi:10.1371/journal.pgph.0002091)

**S2: Abridged anonymised survey responses (does not include free text responses)**


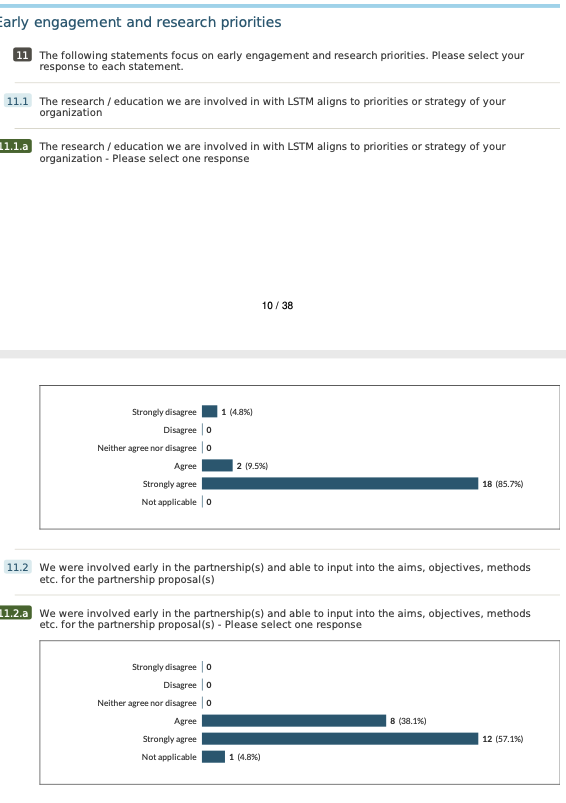


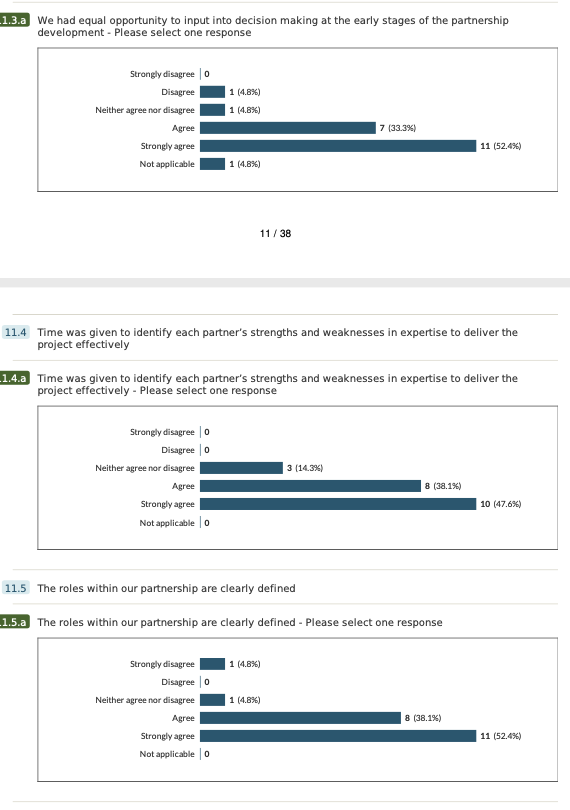


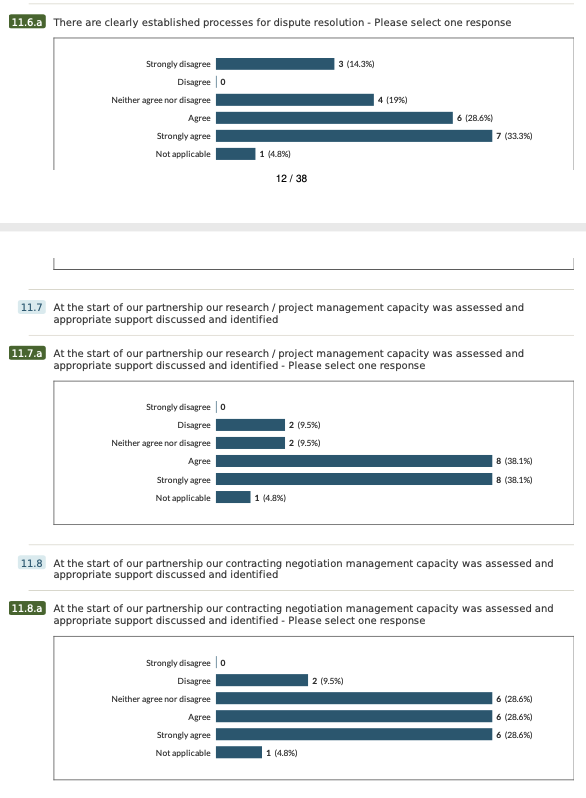


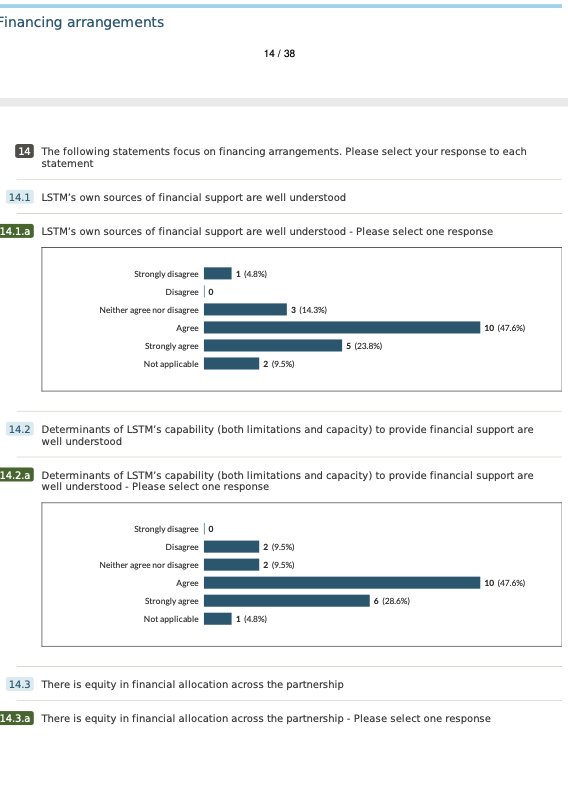


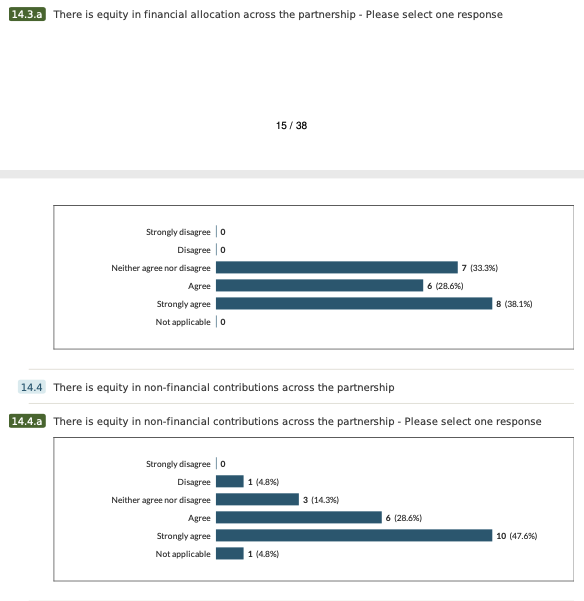


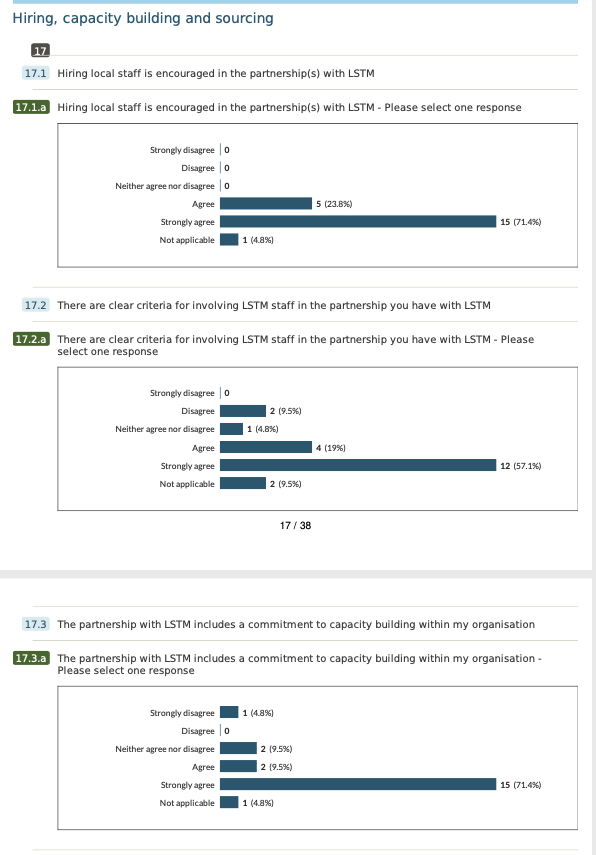


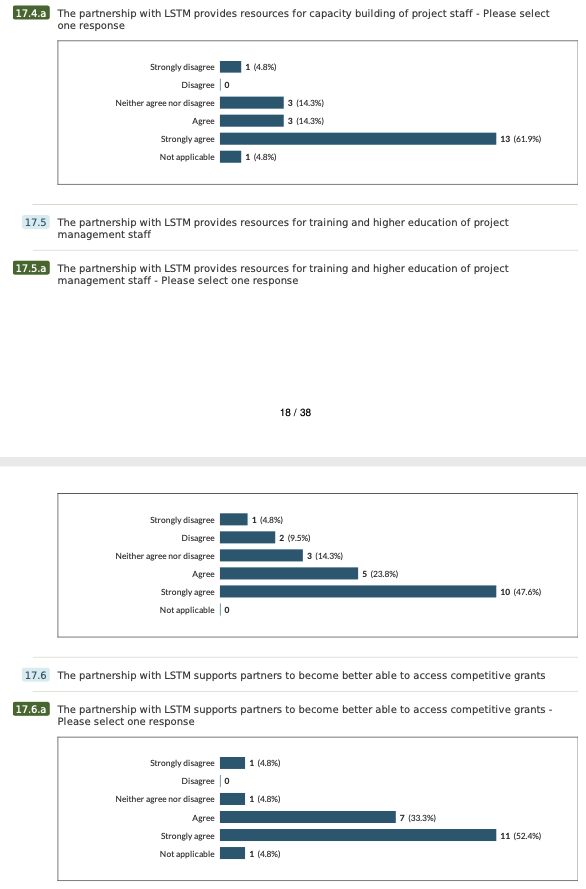


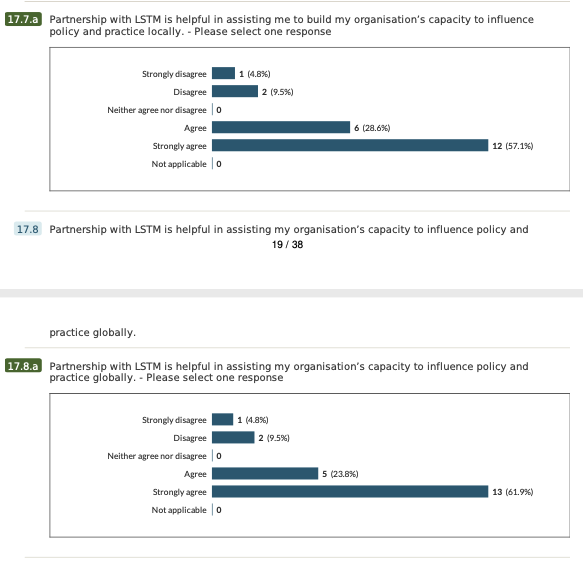


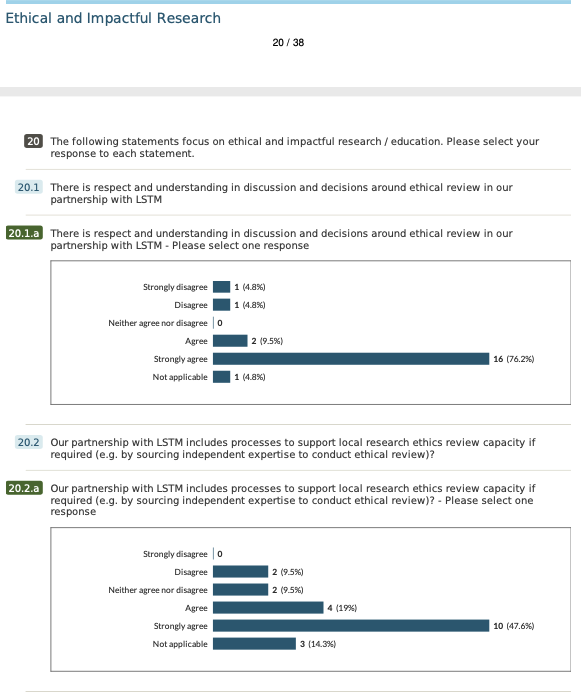


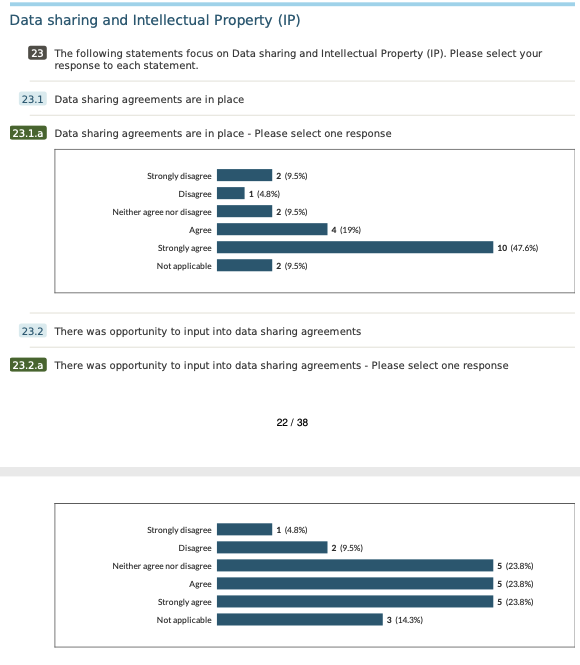


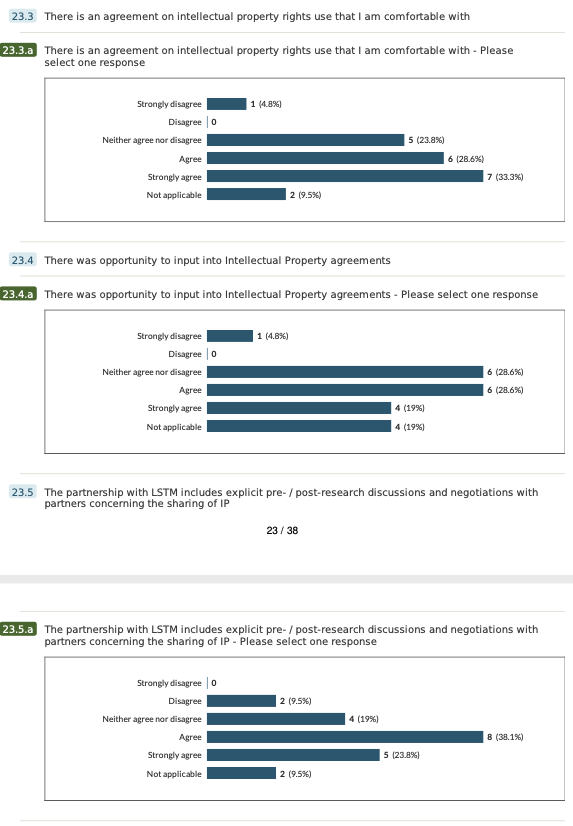


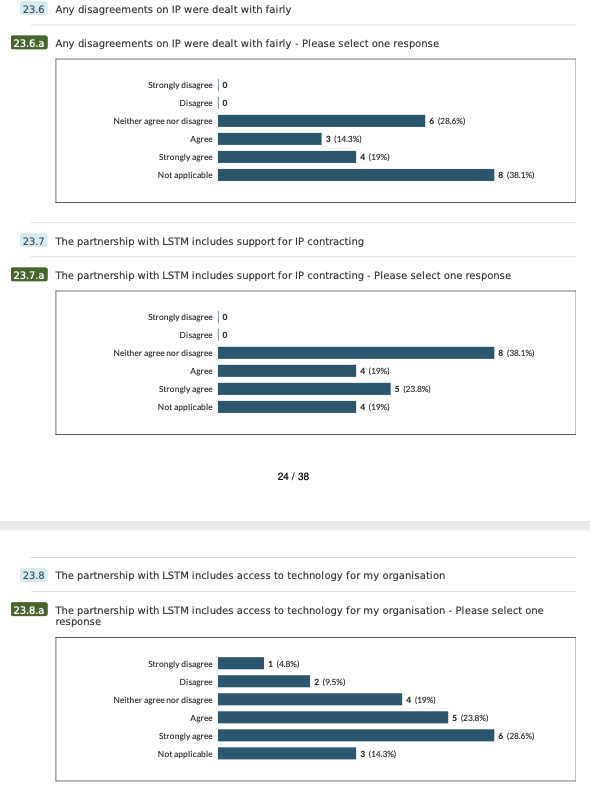


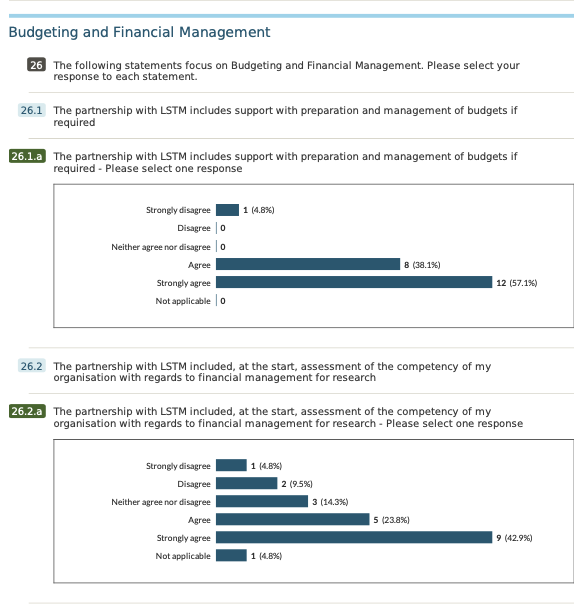


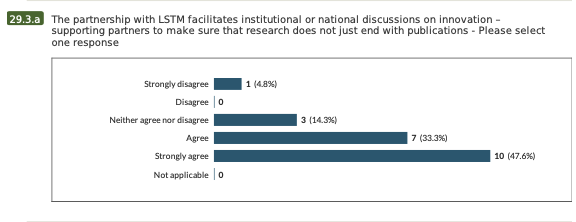


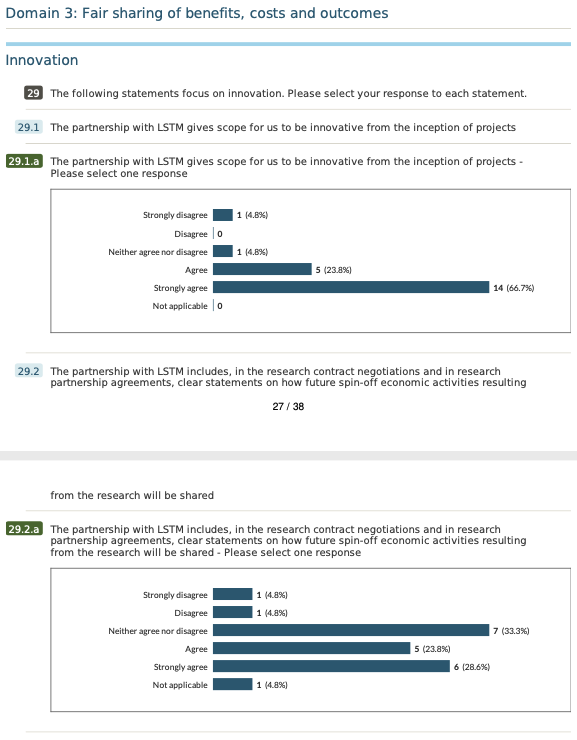


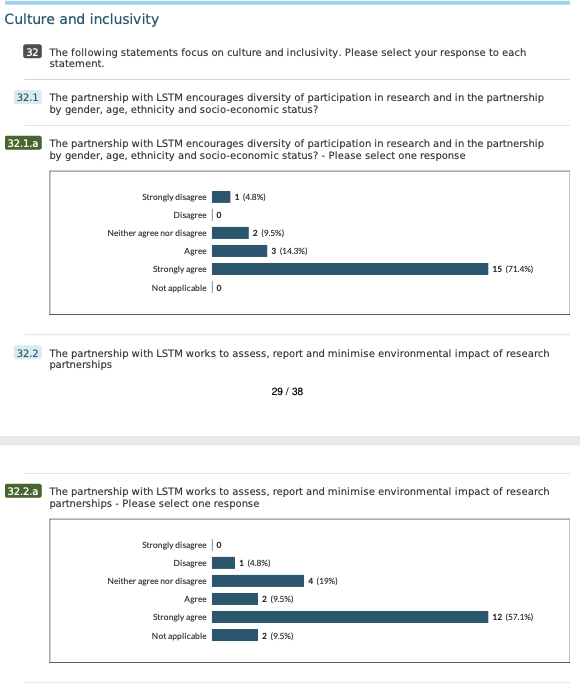


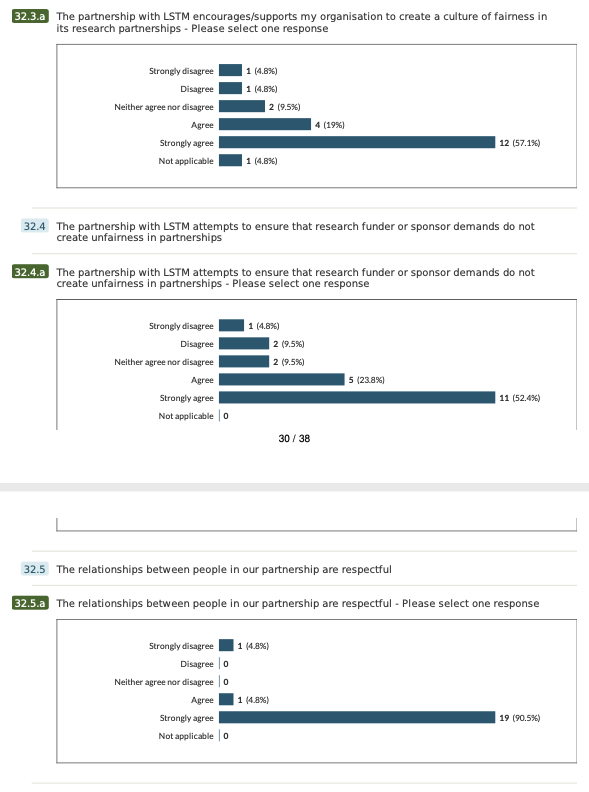


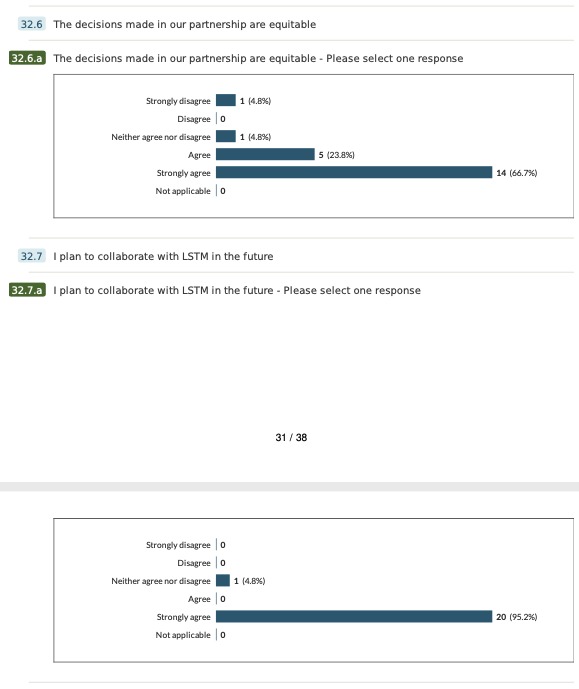


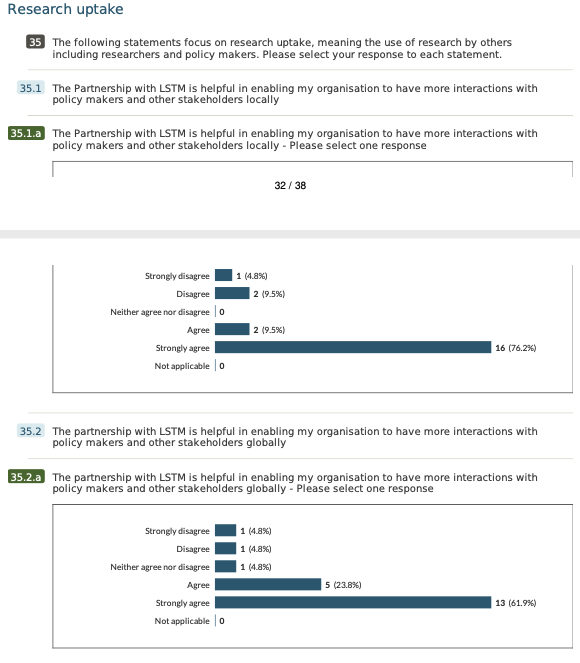


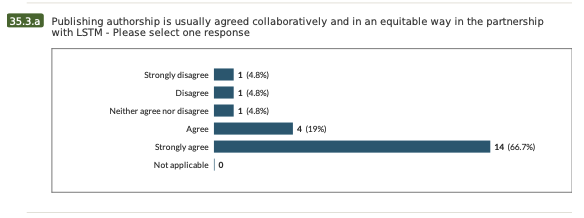


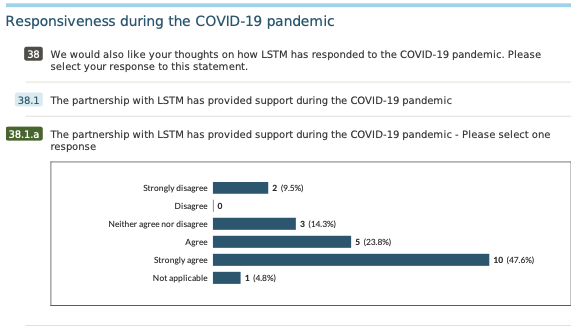

Supplement: S3 Text — (DOCX) [file pgph.0002091.s004.docx]
