# Supplementary material for: A multi-step analysis and co-produced principles to support equitable partnership with Liverpool School of Tropical Medicine, 125 years on
Source: PLOS Glob Public Health. 2024 May 31;4(5):e0002091. doi: 10.1371/journal.pgph.0002091 (PMC11142479; doi:10.1371/journal.pgph.0002091)
Supplement: Attachment — Submitted filename: EP Response to Reviewers 260923ST[89].docx [file pgph.0002091.s005.docx]

Dear Dr Renu Singh,

We thank the reviewers for their helpful critique, which we believe has strengthened the clarity of our findings and helped to situate them in the current context further.

We include point by point responses below.

We also wanted to please understand how joint authorship will work in this paper? Rosie Steege and Robinson Karuga are joint first authors while Sally Theobald and Lillian Otiso are joint last authors.

Please get in touch if we can provide any further information.

Kind regards,

Authorship team

This is a very important topic and you have done a significant amount of work towards developing your co-produced principles. I believe that this paper has promise, but will need some significant revisions.

First, there are some methodological issues where further explanation would be more useful. Also, the paper could be organized more effectively, with more signposting and reference to the principles earlier on. Currently different sections in the paper could be make to flow together more coherently.

We would like to thank you for your thorough review and engagement with this paper. We have signposted to the principles throughout the results to show how the different findings informed each one and also looked at the overall flow for coherence and clarity. We have also provided point by point responses below:

In addition, here are some specific comments to provide you more detail on the methodological questions I had:

1. **Overall, showing more of the data at different phases could be helpful. For example, you could provide the table of partner organizations mentioned in Line 127**.

*We worry that including this table would breech confidentiality of our participants, which we agreed to in the consent process. In order to give as much context as possible whilst maintaining confidentiality we have provided some further information on the organisations in line 122-123.*

**2. I would also discuss further limitations from the various samples/recruitment phases used. For example, Would only working with willing senior leads at LSTM affect the conclusions/potential guidelines that could be included?**

*We believe that this might have influenced the results, and this is discussed within the limitations section of the manuscript. This was the process that we had to negotiate with ethics board to conduct this study so we were limited here but we have brought this reflection up into the methods for transparency – see lines 149.*

**3. The Middle East is mentioned as a region early on (and between the Middle East and North Africa interviews/survey respondents, you do have a few data points here), but then the paper only mentions Africa and Asia. I was wondering why this is the case. It would strengthen the piece to discuss your data for that region as well since it was included in the analysis.**

*Thank you for highlighting this. We had one participant from the Middle East included in the participatory workshop. Their views helped to shape the final principles that emerged. However, we do not have KII or survey data from this region, which is why there is no data included specifically here. We have added this to the limitations section and clarified in our methods – line 155-156.*

**4. For the workshop discussed in Line 155, how many participants were from LMICs? It could help to show the balance between representation from HICs and LMICs through all of the stages of recruitment.**

*We only recruited participants from LMICs. This was in alignment with decolonising agenda and also the fact that the gap in views from LMIC partners were missing from existing principles and guidelines (noted by Voller et al and Monette et al in our refs). This is included in table 1 – but we have further clarified this in the methods on line 170:*

*We held a collaborative workshop in March 2022 with LSTM’s partners working in the global South (n=11) to co-develop a set of principles for equitable partnership (table 1).*

**5. Line 167 discusses the validation exercises, but I do not see a serious discussion and analysis of these inputs.**

*We didn’t have ethics for using comments in the validation in our formal analysis, so we did not use it in analysis. The inputs from the internal and external validation however, informed our thinking, particularly around accountability for these principles. We have reworded this section to avoid over-stating this validation process and clarifying for readers. See line 183 onwards.*

 Reviewer #1: Overall, this is a well-written paper that clearly describes a well-designed and implemented piece of research. The findings and arising discussion points are not novel, but nevertheless contribute to the body of literature on equitable partnership principles and practice by reinforcing themes described elsewhere. Greater reference in the discussion section in particular to the wider literature, including recently published guidelines, would further strengthen the manuscript.

We would like to thank the reviewer for their helpful engagement and constructive review of the paper. We have further engaged with the literature on this topic, which is reflected in an updated discussion. Please see our responses below.

Detailed comments:

**Line 17-18. Confusing to the reader to mention the partners invited to participate in the survey and that the survey was conducted ‘in partnership with LSTM-Liverpool’. Could it instead read ‘the online survey was conducted by LVCT Health and LSTM-Liverpool’ or simply delete this part of the sentence and focus on the respondents whom the survey was aimed at.** -

Thanks for highlighting this, we had meant the survey was exploring those themes in relation to their partnership with LSTM but appreciate this was unclear so have removed this part of the sentence and rephrased.

**Row 41 or 58 Suggest referring to the KFPE Principles (3rd ed) which use the term ‘transboundary’ when introducing the use of this of term in this paper.**

Thank you, we have actioned this on line61

**Row 110-111 Were partners all in LMIC countries, or not? Given core arguments of article, clarity around choice of which partners were contacted would be useful.** Yes, all partners were from LMICs in order to foreground their perspectives in partnership and fill this gap in the literature – we have added text to this effect to clarify, line 112:

“*The exercise was conducted with LSTM-Liverpool’s partners in the global South to foreground their perspectives and co-create principles to strengthen equity. However, these principles are applicable beyond LSTM - to other institutions, research partnerships, and development partners/funders working towards equitable partnerships.”*

**Row 136-7 Would be interesting to know how many organisations were contacted, and to discuss later the potential bias in the results/ reflect on why key informants from these eight but not other institutions responded. This is mentioned in the Limitations section, but it would add transparency to include in the Introduction or Methods the number of institutions contacted in order to see the proportion that responded positively.**

Thanks for highlighting this. This has been added to the methods, we contacted 39 organisations, so you can see the response rate was unfortunately low. Again, we think that this was limited but our ethics procedure that meant we had to get approval from senior heads to cascade down within their organisations, which we now set out in methods for transparency but in more detail in limitations

**Row 139 Since survey responses included individuals from institutions in the Middle East, what was the rationale behind interviewing KIs from Africa and Asia only?**

The respondent from the Middle East participated in our co-development workshop, unfortunately *n*o one from the Middle East was available to participate in the survey or KII. This was reflective of the fewer research partnerships LSTM has in this region but also likely an impact of how the sampling occurred and the strict ethical limitations we had to conduct this research. We have included this in the limitations section to clarify. We have also added a note into methods line 156, and the limitations as follows, see lines 705 onwards.

*“The challenges of recruitment also impacted the global diversity and range of organisations that we spoke with, though our sample included 20 organisations reflective of the areas in which LSTM-Liverpool works. Many organisations who we spoke with were engaged with only a handful of PIs, so the extent to which our findings reflect working partnerships across different departments within LSTM and regions is uncertain. For example, while we had a respondent from the Middle East in the participatory workshop who helped shape the principles, we did not have respondents from this region in our survey or KIIs”*

**Row 167 validation exercise – something a little strange about using LSTM staff to validate findings generated from interviews and survey responses from partner institutions. Implies a sense of hierarchy if LSTM had the opportunity to moderate what was said by partners, rather than the other way around, for example. Suggest adding a bit more explanation about the rationale underpinning this step.**

Thank you for bringing this to our attention – it is important for us to clarify; our first step was to validate our findings from the KIIs and survey with the partners during our co-development workshop. Then we took these findings and the co-developed principles that came out of that workshop to share with LSTM staff to see if these findings resonated. There was a conference in light of LSTM’s 125 anniversary, so it was a timely opportunity to share what we had been working on and stimulate reflection among staff, but this was not about moderating the findings. We then repeated this process by presenting the same findings externally at HSG Bogota, to again get a sense of whether these findings resonated with a broader audience. This raised new questions around accountability, which are laid out in our discussion.

We have edited this process for clarity on lines 171-177

**Row 196. What about written or verbal consent for participation in interviews?**

Thank you, for spotting this error – it is now rewritten:

This study received approval from the Research Ethics Committee at LSTM (21-060) in September 2021. Participant information sheets were provided, and written and/or verbal informed consent was obtained for all survey responses, KIIs and workshop participants, please see Lines 206-208.

**Row 211 Table 1. Use different term from ‘nationality’ as this is misleading, since the respondents nationalities are grouped into regions.**

Thank you for spotting this, we have rephrased to ‘Geographical Region’ in table 1.

**Row 229 and other quotes where ‘Senior/ ECR’ is used. Need to spell out acronym ‘ECR’ at first use, and also include seniority in Table 1 as one of the demographic indicators by which respondents were categorized. Also, why categorise KIs but not survey respondents? And what were the cut-offs for whether someone was Senior or an ECR?**

We have now spelt out ECR on 1^st^ mention. We have not categorised the survey respondents as the survey was anonymous and not all participants provided this information, which is why it is also not reflected in the table. For the interviews, the title was self-selecting, i.e. no formal cut off, but we asked respondents how they identify themselves. We wanted to keep the range because, they will have different experiences and views in regard to the topic, for example the views of ECRS included emphasis on capacity strengthening in partnerships.

**Row 427. How are you differentiating between ‘fair’ and ‘equitable’? I see the terms as synonymous, and as such, using both in the same sentence is duplicative. If the authors identify a distinction, worth articulating this.**

We agree and have reworded to read equitable only

**Row 451-453. Suggest a shorter description of Fig 2 – something like ‘Research-derived values of equitable partnership’ and bringing up the description of the steps towards identifying and verifying the principles into rows 451-453**.

Thank you for this, we have now reedited as per your suggestion –lines 458 – 467.

**Row 463 Include reference to the TRUST Global Code of Conduct.** – Thank you, this is now included

**Row 476 Discussion section should add more references to other recent work which has elicited very similar findings and discussion points. Most of the themes presented are not original. This is not a problem since the study generates additional supporting evidence of the importance of these themes, but it seems an omission not to contextualise the study alongside other similar work**

Thank you for this suggestion, we have now added recent literature to reflect this throughout the discussion in tracked changes.

**Row 476 In the Discussion, there is not much discussion of the conflict faced by HIC institutions whereby fully embracing the principles of equitable partnership to some extent inhibits individual staff and institutional success, e.g authorship, allocation of funds etc. Worth putting this contentious challenge on the table more explicitly?**

Thank you for this important reflection, which we have discussed and grappled with ourselves. We agree that it is important to be explicit about this so we have now added these issues and challenges under the section of what LSTM needs to do – and included the link to principle 5.

**Row 525 Be explicit about which UKCDR definition is being referred to – presumably their definition of equitable partnerships? Include reference to UKCDR website/source document. Spell out acronym at this first use.**

We have actioned this.

**Row 649. Why a need to engage with more junior researchers but not research administrators/ managers? Were professional staff included as respondents? If so (or if not) specify in Methods section and if not, include this as a limitation since professional staff are a critical stakeholder group.**

We agree this is a critical limitation and an area for future research. While some professional services staff may have been included in the survey the anonymous nature means we cannot confirm this. The ethical agreement we had negotiated to conduct this study, meant that we had to go through senior or heads of organisations and ask them to cascade internally. This meant that the power hierarchies that exist in research are reflected in our sampling. We have now more critically discussed this in the limitations.

**Minor points on spelling, grammar and style** -

- **Row 110. ‘Across 15 different country contexts…’. Why not just say ‘across 15 countries…’? Similar comment applies in row 136. We have actioned all instances**
- **Row 343 A minor semantic point, but ‘virtual mediums’ sounds rather psychic. Could ‘virtual platforms’ be a better term? Agree! We have reworded**
- **Row 388 and 393 as examples. Check consistency of US v. UK spelling of words such as organization/ organisation. And Upper v lower case, e.g. row 430 v 439 northern/ Northern We have checked this**
- **Row 441 Use brackets around description of respondent. Also be consistent in whether respondent descriptor is written in italics or not. Thank you for spotting, corrected**
- **Row 482 Commas in the wrong place. Should read ‘Research partnerships were, overall, viewed positively and equitably. Corrected**
- **Row 594 (and elsewhere) BMJ – spell out acronym at first use. Checked and corrected**
