# Supplementary material for: A multi-step analysis and co-produced principles to support equitable partnership with Liverpool School of Tropical Medicine, 125 years on
Source: PLOS Glob Public Health. 2024 May 31;4(5):e0002091. doi: 10.1371/journal.pgph.0002091 (PMC11142479; doi:10.1371/journal.pgph.0002091)
Supplement: Attachment — Submitted filename: Plos GH Review.docx [file pgph.0002091.s006.docx]

Review: A multi-step analysis and co-produced principles to support Equitable Partnership with Liverpool School of Tropical Medicine, PGPH-D-23-00801

This is a critical topic; seeing these types of studies is excellent. Overall, it does not feel like the paper itself ‘is walking the walk’, and I would love to explore a few possible ways to help think further about the *praxis* of this work. Overall, there needs to be more nuanced questioning in the form of “who was not at the table” in every aspect of writing this paper.

1. **Epistemic lens** – The importance of epistemic diversity is mentioned (line 80), but this paper does not feel like it *owns* that principle in its own writing. The importance of shifting power feels like it might also be helpful to do that with the frameworks in which we write. Though the application of the RFI (Research Fairness Initiative) framework fits well here, there should be additional mentions of other frameworks derived with diverse epistemic understandings. For example, I would have loved to see a framework around collectivism or polychronic time, both often values in LMIC contexts that get disregarded with global partnerships. To strengthen this paper, it would be great to discuss some additional frameworks, why they were not used, and/or if some elements of non-Western derived frameworks could also be analyzed. Also diversify the references to include more local work.
2. **Positionality statement** – There are a lot of limitations of this study, which are well noted at the end. At the end of the paper, it was noted that some journals are helping with academic fairness by including ‘reflexivity statements.’ This paper could be significantly strengthened with a positionality statement for each author (or as a group) describing worldviews and proximity to power. This can help set the tone of the paper, also recognizing that this author group represents certain world views, positionality and proximity to power that makes this work only generalizable to people and populations who share such worldviews. This builds off the theme noted—specificity is critical and avoiding the over-homogenizing stereotype(s).
3. **Definition of ‘Partner’**- It would be really helpful to have a definition of “Partner” and “Partnership” at the beginning of the paper. It feels critical to understand how the institution and author groups define partners, as this could also add to colonial structures. As this paper illustrates, how partnerships work can create and be a product of structural violence, so using defined (and pre-existing) partnerships may be perpetuating “good feedback” bias, as these partners may also have ‘relative’ elite status and proximity to power that needs to be acknowledged.
4. **Define ‘varied respondent’** – It needs to be more clear who was responding to KIIs. Please define exactly who was a varied respondent.
5. **Address lack of central and S. America partnerships**- This was a limitation of the study, there is a lack of geographic diversity. There are also no partners from Eastern Europe. If this is the case- critically explore why not?
6. “**Data was validated”** – Line 84- needs to be more detailed. Validated by what tool? Methodology here is lacking.
7. **Personality differences**- The discussion/theme around the importance of personality differences in PIs and its impact on partnerships is wonderful. This is addresses the critical importance of interpersonal relationships in this work. This should be more fully discussed in the discussion around accountability metrics.
8. **Line 433-435 discussed the difference between academic and non-academic partnerships**. This was a great point- but should also be more interrogated in the discussion with an accountability plan. Why is it that academics are more interested in outcomes? What colonial structures allow for non-academic partnerships to be more flexible?
9. **Accountability**- This paper needs more discussion around accountability, reparations, and what the institution owes to its ‘partners.’ These principles that were generated are great- but in order to not add more noise to the decoloniality literature, this paper needs to own some elements of accountability to change.
