# Supplementary material for: A multi-step analysis and co-produced principles to support equitable partnership with Liverpool School of Tropical Medicine, 125 years on
Source: PLOS Glob Public Health. 2024 May 31;4(5):e0002091. doi: 10.1371/journal.pgph.0002091 (PMC11142479; doi:10.1371/journal.pgph.0002091)
Supplement: Attachment — Submitted filename: Response to Reviewers 150324.docx [file pgph.0002091.s007.docx]

15.03.2024

Dear Dr Heather Haq,

**Re: A multi-step analysis and co-produced principles to support Equitable Partnership with Liverpool School of Tropical Medicine, PGPH-D-23-00801**

We would like to thank the reviewer for their thoughtful, insightful critique, which we believe will further strengthen the paper, and as noted, make it more useful in practice. We include point by point responses below.

Kind regards,

The authorship team

This is a critical topic; seeing these types of studies is excellent. Overall, it does not feel like the paper itself ‘is walking the walk’, and I would love to explore a few possible ways to help think further about the *praxis* of this work. Overall, there needs to be more nuanced questioning in the form of “who was not at the table” in every aspect of writing this paper.

We do agree with this analysis and would like to thank you for your helpful and insightful comments, which we have addressed below and signposted to the manuscript. In particular, we have included more critical reflections on who LSTM’s partners are, and who was therefore not included in the development of our principles, and who was not part of the process. To note, we see this work as a starting point, or indeed a critical entry point to a joint inquiry to more transformative relationships (Aboderin et al. 2023) and will continue to reflect on this work with our partners as these partnerships evolve through time.

1. **Epistemic lens** – The importance of epistemic diversity is mentioned (line 80), but this paper does not feel like it *owns* that principle in its own writing. The importance of shifting power feels like it might also be helpful to do that with the frameworks in which we write. Though the application of the RFI (Research Fairness Initiative) framework fits well here, there should be additional mentions of other frameworks derived with diverse epistemic understandings. For example, I would have loved to see a framework around collectivism or polychronic time, both often values in LMIC contexts that get disregarded with global partnerships. To strengthen this paper, it would be great to discuss some additional frameworks, why they were not used, and/or if some elements of non-Western derived frameworks could also be analyzed. Also diversify the references to include more local work.

Thank you for highlighting this, we do agree that over reliance on non-Western frameworks is a limitation here. However, non-western input into existing guidance around equitable partnerships was a key critique of ours and indeed a rationale for doing this work. In a 2022 review only 2//22 guidance documents and references for equitable partnerships, were developed by or with Southern Partners. Our goal in using the RFI was to use something existing to structure our discussions with partners and add their perspectives to. This is a fast-moving field and at the point of developing the study we could not find Southern frameworks that spoke specifically to partnerships at the time of developing this study. We first submitted this paper in May 2023 – since then, there have been many more Southern led publications on this topic, which is excellent, but not all of these were available at the time the study was conceptualized in 2021. We have now included additional literature here including for example Aboderin et al (2023) and Aubel and Chibanda (2022) which discuss transformative partnerships and the importance of culture respectively.

The concept of Polychronic time and collectivism are interesting and although certainly applies to our partners, did not emerge explicitly in our findings.

2. **Positionality statement** – There are a lot of limitations of this study, which are well noted at the end. At the end of the paper, it was noted that some journals are helping with academic fairness by including ‘reflexivity statements.’ This paper could be significantly strengthened with a positionality statement for each author (or as a group) describing worldviews and proximity to power. This can help set the tone of the paper, also recognizing that this author group represents certain world views, positionality and proximity to power that makes this work only generalizable to people and populations who share such worldviews. This builds off the theme noted—specificity is critical and avoiding the over-homogenizing stereotype(s).

We developed a reflexivity statement as we wanted to model this (see appendix 4). This was structured based on Morton et al. In response to your helpful comments, we have added additional text on our group positionality statement to the section on positionality, power and engagement within the text, see line 221 onwards.

3. **Definition of ‘Partner’**- It would be really helpful to have a definition of “Partner” and “Partnership” at the beginning of the paper. It feels critical to understand how the institution and author groups define partners, as this could also add to colonial structures. As this paper illustrates, how partnerships work can create and be a product of structural violence, so using defined (and pre-existing) partnerships may be perpetuating “good feedback” bias, as these partners may also have ‘relative’ elite status and proximity to power that needs to be acknowledged.

Thank you for this critical reflection – we do agree with you on the nature of using pre-existing partnerships and it has been something we have been reflecting on as an institution since we submitted this paper originally. We have now included further reflection on this in the limitations section with a stronger and more critical reflection on who was “at the table” and the implications of this.

4. **Define ‘varied respondent’** – It needs to be more clear who was responding to KIIs. Please define exactly who was a varied respondent.

We have edited this image and added a reference to table 1 which gives details on who responded to KIIs.

5. **Address lack of central and S. America partnerships**- This was a limitation of the study, there is a lack of geographic diversity. There are also no partners from Eastern Europe. If this is the case- critically explore why not?

Thank you for these insightful reflections, we have added further detail in the limitations section on who was included in the selection process, and who was excluded, and how this reflects history and the implications of exclusions of our analysis process (lines 728-737). To note again, this piece of work was designed as an entry point and is ongoing within our institution. We have also prefaced this upfront in our discussion section – see lines 499 -504.

6. “**Data was validated”** – Line 84- needs to be more detailed. Validated by what tool? Methodology here is lacking.

We had used the term validation to mean we affirmed our analysis via participant checking – we have now changed the language to ‘a participant and wider stakeholder checking process’.

7. **Personality differences**- The discussion/theme around the importance of personality differences in PIs and its impact on partnerships is wonderful. This is addresses the critical importance of interpersonal relationships in this work. This should be more fully discussed in the discussion around accountability metrics.

Thank you, we have added some ideas around accountability metrics here into the discussion. Line 682 onwards.

8. **Line 433-435 discussed the difference between academic and non-academic partnerships**. This was a great point- but should also be more interrogated in the discussion with an accountability plan. Why is it that academics are more interested in outcomes? What colonial structures allow for non-academic partnerships to be more flexible?

Thank you, we have added this to the discussion lines 697-703.

9. **Accountability**- This paper needs more discussion around accountability, reparations, and what the institution owes to its ‘partners.’ These principles that were generated are great- but in order to not add more noise to the decoloniality literature, this paper needs to own some elements of accountability to change.

Yes, we do agree and have restructured and updated the section on what LSTM is doing and we are thinking critically about different accountability measures. Since many months have passed, we have begun actioning changes to work towards more equitable/transformative partnerships – including through a partnership council and our research integrity working group, this is occurring alongside decolonisation work that is not discussed in the remit of this paper. We have written this in the discussion – page 27.
